# Thermal Variability Increases the Impact of Autumnal Warming and Drives Metabolic Depression in an Overwintering Butterfly

**DOI:** 10.1371/journal.pone.0034470

**Published:** 2012-03-30

**Authors:** Caroline M. Williams, Katie E. Marshall, Heath A. MacMillan, Jason D. K. Dzurisin, Jessica J. Hellmann, Brent J. Sinclair

**Affiliations:** 1 Department of Biology, University of Western Ontario, London, Ontario, Canada; 2 Department of Biological Sciences, University of Notre Dame, South Bend, Indiana, United States of America; University of Sao Paulo, Brazil

## Abstract

Increases in thermal variability elevate metabolic rate due to Jensen's inequality, and increased metabolic rate decreases the fitness of dormant ectotherms by increasing consumption of stored energy reserves. Theory predicts that ectotherms should respond to increased thermal variability by lowering the thermal sensitivity of metabolism, which will reduce the impact of the warm portion of thermal variability. We examined the thermal sensitivity of metabolic rate of overwintering *Erynnis propertius* (Lepidoptera: Hesperiidae) larvae from a stable or variable environment reared in the laboratory in a reciprocal common garden design, and used these data to model energy use during the winters of 1973–2010 using meteorological data to predict the energetic outcomes of metabolic compensation and phenological shifts. Larvae that experienced variable temperatures had decreased thermal sensitivity of metabolic rate, and were larger than those reared at stable temperatures, which could partially compensate for the increased energetic demands. Even with depressed thermal sensitivity, the variable environment was more energy-demanding than the stable, with the majority of this demand occurring in autumn. Autumn phenology changes thus had disproportionate influence on energy consumption in variable environments, and variable-reared larvae were most susceptible to overwinter energy drain. Therefore the energetic impacts of the timing of entry into winter dormancy will strongly influence ectotherm fitness in northern temperate environments. We conclude that thermal variability drives the expression of metabolic suppression in this species; that phenological shifts will have a greater impact on ectotherms in variable thermal environments; and that *E. propertius* will be more sensitive to shifts in phenology in autumn than in spring. This suggests that increases in overwinter thermal variability and/or extended, warm autumns, will negatively impact all non-feeding dormant ectotherms which lack the ability to suppress their overwinter metabolic thermal sensitivity.

## Introduction

The intuitive outcome of warmer winter temperatures for ectotherms is a release from cold-induced stress, leading to increased survival and expansion of geographic ranges [1], [2]. However, metabolic rate is a function of temperature in many species, such that warmer winters result in individuals emerging from dormancy in spring with decreased energy reserves [3], reducing energy available for post-winter maintenance and reproduction, thereby depressing performance and fitness [4]. In holometabolous insects the negative consequences of energetic depletion may be particularly pronounced, as energy reserves derived from larvae supply resources for vitellogenesis and somatic maintenance [5], and some essential nutrients are derived solely from the larval diet [6]. Temperate univoltine insects are often dormant from autumn to spring (the ‘overwintering period’) in late juvenile life stages, with limited or no opportunity to feed before metamorphosing into adults in the spring, making them potentially vulnerable to energetic drain as a result of changing winters. Here, we explore the impact of thermal variability on overwinter energy use in a butterfly larva in the context of climate change.

**Figure 1:**
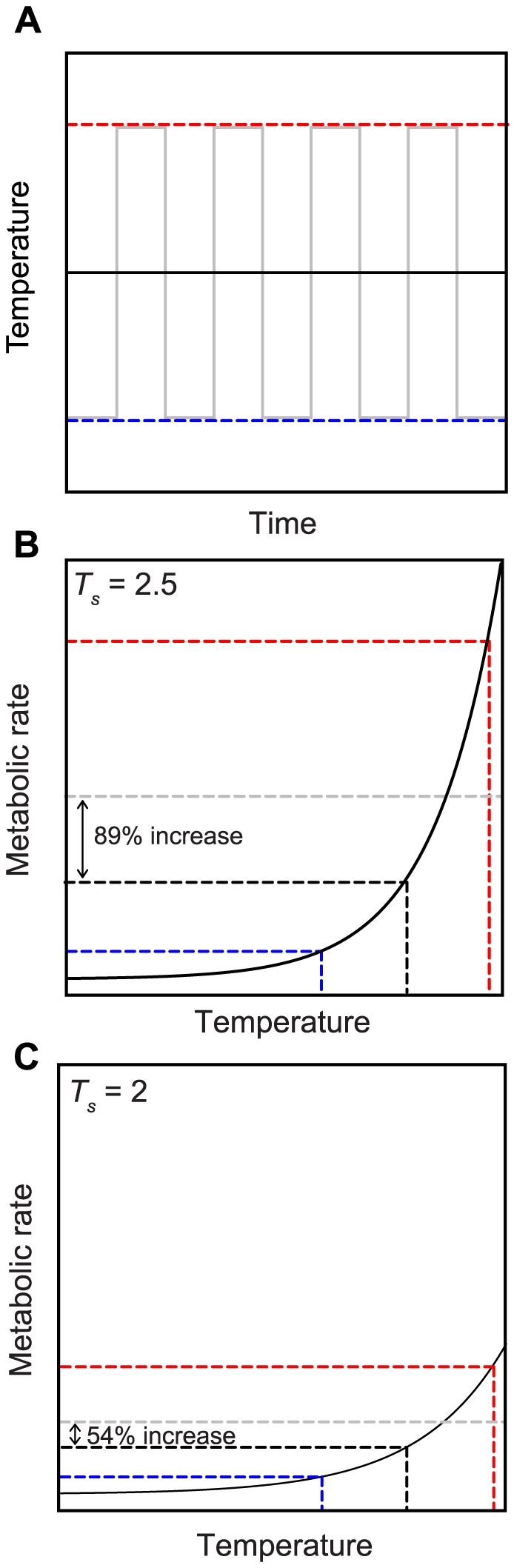
The effects of Jensen's inequality and thermal sensitivity on ectotherm metabolic rate under fluctuating temperatures. (A) Hypothetical constant (black) and fluctuating (gray) temperature regimes. The high and low points of the fluctuating regime are marked by the dotted red and blue lines respectively. (B) The accelerating portion of a hypothetical metabolic rate-temperature curve (equation 1) with a thermal sensitivity (*T_s_*) of 2.5, with the high and low points of the fluctuating regime marked in red and blue doted lines as in (A), while the constant regime is indicated by the black dotted line. The gray dotted line shows the mean value of metabolic rate under the fluctuating regime, which is 89% higher than the mean value of metabolic rate under the constant regime. (C) The same curve (from equation 1) but with lower thermal sensitivity (*T_s_* = 2), showing that the increase in mean metabolic rate imposed by thermal variability is reduced to 54% when thermal sensitivity is decreased compared to (B). *T_s_* is the only parameter that changes between (B) and (C), as the scaling factor (*S* from equation 1) and axes are held constant.

It is well-appreciated that an acute increase in temperature will result in an increase in the metabolic rate of an ectotherm. Metabolic rate has a curvilinear relationship with temperature in ectotherms, typically accelerating to an inflection point well below the thermal optimum and then decelerating until the thermal optimum is reached, after which it declines sharply until critical thermal limits are exceeded and death occurs [7]. Thermal variability can also have a significant impact on energy consumption by ectotherms. Jensen's inequality states that the mean value of metabolic rate over the accelerating portion of the curve will increase with increasing variance in temperature [8]. Thus, the warm part of daily thermal cycles will disproportionately increase metabolic rate beyond what would be predicted by mean temperatures (Fig. 1). The magnitude of this effect will depend on the degree of curvature (the thermal sensitivity) of the rate-temperature function over the range of temperature fluctuations, as well as the magnitude of the temperature fluctuations (Fig. 1C; [9]). In an exponential model of temperature-metabolic rate relationship (where *B* is metabolic rate, *S* is a scaling factor, and *T* is temperature)
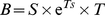
(1)thermal sensitivity is represented by the exponent *T_s_*, which is similar to Q_10_ (or the ‘Boltzmann factor’ in the metabolic theory of ecology [10]). Jensen's inequality underlies the sensitivity of tropical organisms to temperature increases [11], and can explain discrepancies between experiments conducted at fluctuating and constant temperatures (e.g. [12]–[14]). It is important to note that although some metric of thermal sensitivity of metabolism is essential to any model of metabolism, it cannot be predicted from first principles, and is thus generally assumed or must be estimated from empirical data [15].

Diapausing temperate insects may modify their overwinter energy use by an overall suppression of metabolism (a lower elevation of the temperature-metabolic rate curve) and also by modifying the shape of the curve, thus changing the thermal sensitivity of metabolic rate [4]. A suppressed metabolic rate across the thermal range experienced decreases overall rates of energy use [4], but a reduced thermal sensitivity of metabolic rate has the potential to lessen the metabolic impact of variability by reducing the relative impact of the warm portions of thermal cycles [8]. As successful overwintering demands the conservation of stored energy reserves, low thermal sensitivity could thus increase fitness of overwintering insects in thermally variable environments. If there is heritable variation in the degree of thermal sensitivity of metabolism, then energetically-demanding (warm or thermal variable) environments would be expected to select for depressed thermal sensitivity in populations from such environments. There does appear to be heritable variation in those metabolic traits measured thus far in ectotherms (e.g. [16]), although, to our knowledge, the heritability of thermal sensitivity itself has not yet been assessed. Temporally heterogeneous environments are theoretically predicted to decrease the thermal sensitivity of performance functions [8], but this has not yet been empirically supported with regards to variability on a daily timescale [17].

The timing of entry into and exit from dormancy is also a powerful determinant of energy used, and interacts with characteristics of the thermal environment. Both an earlier entry into and later exit from dormancy will increase the total amount of energy used by increasing the period of time spent dormant. Indeed, longer periods of dormancy do increase energy drain in some insects [4]. Even if the length of dormancy remains constant, phenology has the potential to impact overwintering energetics due to the different thermal profiles of autumn (which is relatively warm) and spring (relatively cool). Thus, extended pre-winter dormancy reduces fitness in insects [18], [19], presumably due to the impact of warm autumn temperatures on metabolic rate during this period. To date, no models exist exploring the energetic consequences of changes in phenology, and the interaction between phenology shifts and thermal variability.

Here, we examine the impact of thermal variability on overwinter energy use by larvae of *Erynnis propertius* (Lepidoptera: Hesperiidae), and how phenological shifts might alter energy use in stable compared to variable environments. *Erynnis propertius* larvae in the Pacific Northwest feed on oak (*Quercus* sp.) in the summer, overwinter as a dormant sixth instar larva in the leaf litter, and do not feed again before metamorphosis and adult flight in spring [20]. This species overwinters in both thermally-stable and -variable environments, and we reared individuals in a reciprocal common-garden experiment with stable and variable thermal regimes, allowing us to disentangle the genetic and maternal effects of origin from the phenotypic responses to thermal variability. We expect that *E. propertius* provides a generalisable model for non-feeding dormant ectotherms occupying environments of varying thermal variability, and also those thrust into changing variability through climate change or geographic range expansion. We first measured the impact of these regimes on the thermal sensitivity of metabolic rate, testing the hypothesis that a high daily thermal range would elicit a decrease in the thermal sensitivity of overwintering insects, in order to compensate for the increased energetic demands of thermally variable environments. We then used this information to explore the impact of this thermal sensitivity on overwinter energy use in *E. propertius* larvae in a model predicting overwinter energy use over 27 years, and used this model to explore the energetic impacts of phenological shifts. We show that winter metabolic rate is suppressed by variable environments, and that most of the energy used during the dormant period is consumed in the autumn. Thus, shifts in autumn conditions and phenology may be critical in the impact of climate change on other non-feeding overwintering ectotherms in temperate regions.

## Materials and Methods

### Study animals and rearing

All necessary permissions were obtained for the field components of this work. Collection and access permission was obtained in Canada from Capital Region District Parks (permit VI0310074), the Department of National Defense (P090-09), and via a letter of understanding from the Nature Conservancy of Canada. Collection and access permission was obtained in Oregon from a letter of understanding from a private landowner, and via a letter of understanding from the Bureau of Land Management.

**Figure 2:**
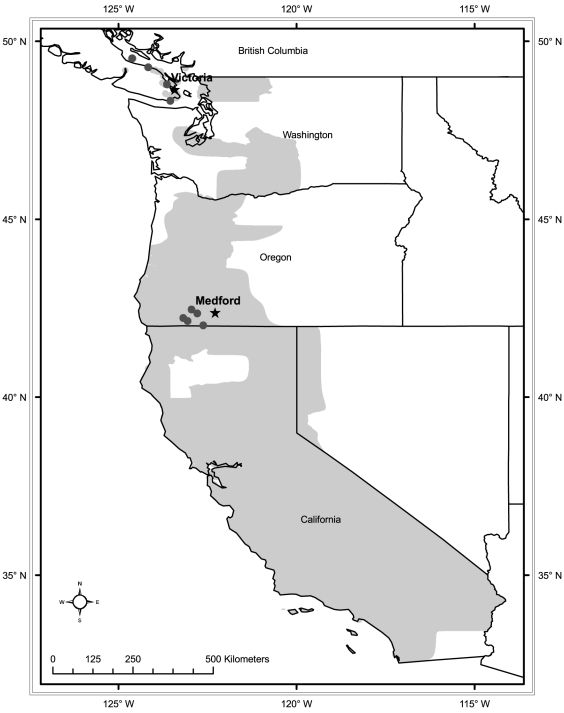
Collection sites and the geographic range of *Erynnis propertius*. Butterflies were collected from southern Vancouver Island, British Columbia and the vicinity of Medford, Oregon, overall distribution of this species is shaded in grey. Circles indicate the sampling locales and stars the location of the climate stations from which meteorological data were obtained. *E. propertius* has also been reported from Baja California Norte, Mexico (not shown). Data from Opler PA, Lotts K, Naberhaus T (coordinators) (2011) *Butterflies and Moths of North America*. http://www.butterfliesandmoths.org/; and Royal British Columbia Museum Entomology Collection, Canadian National Collection (CNC) of Insects, Arachnids and Nematodes, Lyman Entomological Museum, Nova Scotia Museum of Natural History, Halifax, NS, Canada, Lepidopterists Society Season Summaries 1973–1997, Crispin S. Guppy Collection, Royal Ontario Museum: Entomology, and the Spencer Entomological Museum (accessed through GBIF Data Portal, data.gbif.org).

**Figure 3:**
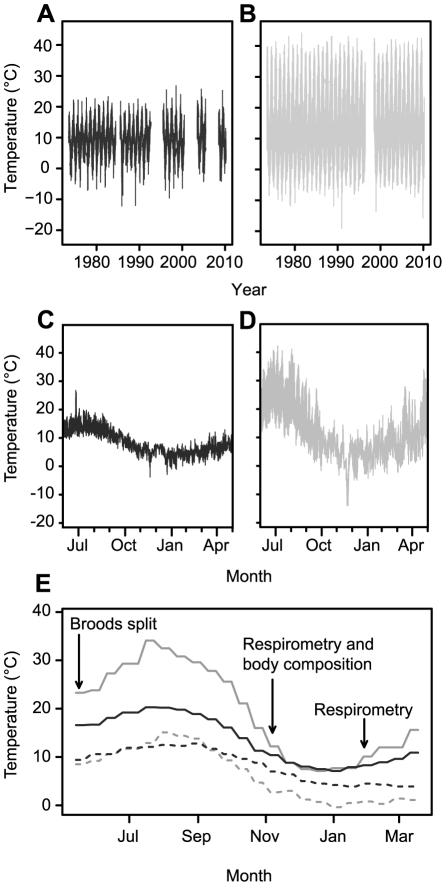
Temperature records from Vancouver Island and Oregon. Hourly meteorological data from (a) Victoria International airport, BC, 1973–2010; (b) Victoria International airport, BC, 1998–1999; (c) Rogue Valley International-Medford, OR, 1973–2009; (d) Rogue Valley International-Medford, OR, 1998–1999; (e) normal-based incubator temperature regimes, with timing of experimental events indicated.

*Erynnis propertius* eggs were collected from fertilised females caught in Garry Oak (*Q. garryana*) savannahs on Vancouver Island, British Columbia (BC populations) and near Medford, Oregon (OR populations; Fig. 2) in April–May 2009. Eggs were shipped to the University of Notre Dame, and larvae were raised in growth chambers on fresh-cut Garry Oak leaves on a 12h∶12h (light∶dark) thermo- and photoperiod (Fig. 3; [21]). Temperature regimes were based on climate normals from nearby meteorological stations for Victoria, British Columbia (BC) and Medford, Oregon (OR). BC has a relatively low-variability thermal environment, referred to as ‘stable,’ while the climate in OR fluctuates widely, referred to as ‘variable’ (Fig. 3, Table 1). Broods were split so that offspring from each mother were represented in each rearing condition, resulting in four treatment groups (geographic origin/rearing conditions): 1) BC/stable; 2) BC/variable; 3) OR/stable; and 4) OR/variable. In late August 2009 larvae were transferred to the University of Western Ontario and kept in Sanyo MIR-153 incubators (Sanyo Scientific, Bensenville, IL, USA) with *ad libitum* fresh Garry Oak leaves until the caterpillars entered dormancy. A larva was considered dormant when it was brown, ceased feeding for three days, and was rolled in a leaf. Dormant larvae were removed from leaf rolls, and transferred to 6-well plates with a moist paper towel to maintain high humidity, in constant darkness to approximate conditions in a leaf roll underneath leaf litter.

**Table 1:** Summary of climate data from Vancouver Island and Oregon.

	BC	OR
**Location of weather station**	48.38°N, 123.25°W	42.23°N, 122.52°W
**Elevation**	19 m	405 m
**Coverage**	1973–2010	1973–2010
**Missing winters**	1984, 1992–1994, 2000–2002, 2005–2007	1996–1997
**Annual mean temperature (°C)**	8.6±3.9	12.3±9.1
**Mean temperature during dormancy (°C)**	7.9±3.4	10.0±8.4
**Annual mean daily range (°C)**	3.7±0.9	14.1±4.42
**Absolute maximum (°C)**	26.8	44.4
**Absolute minimum (°C)**	−12.2	−19.4

Meteorological data are from the climate monitoring stations closest to the study locations (Fig. 2). Procedures for excluding meteorological data are described in the methods. Temperature during dormancy is the mean temperature between 12 August and 23 March each year; the approximate dates over which larvae are dormant at both locations. Mean ± SD is given where appropriate.

### Physiological measurements

In November, after all individuals had entered dormancy, larvae were weighed (Mettler-Toledo MX5, Columbus, OH, USA; d = 0.1 µg) and five larvae per treatment group were snap-frozen in liquid nitrogen and stored at −80°C for lipid quantification by thin layer chromatography coupled to a flame ionisation detector (TLC-FID) using 1-stearoyl-rac-glycerol as an internal standard (Sigma-Aldrich, St Louis, MO, USA) and a benzene∶ chloroform∶ formic acid (70∶30∶0.5 v/v/v) solvent system [22]. Lipid concentration was expressed for each individual per unit dry mass of tissue. Total lipid content was estimated from mean lipid concentrations and mean dry mass of source×rearing treatment combinations (groups were pooled if not significantly different), assuming 75% water content for this species [22].

Carbon dioxide production was measured using flow-through respirometry [23] in six larvae from each treatment group, each at 10, 15, 20, 25, and 30°C (November) or 1, 8, 18, 25, and 30°C (February) in randomised order at a randomised time of day between 0800 and 1800 with at least three days between measurements for any one individual. Dry, CO_2_-free air was passed through an 11 cm^3^ chamber containing a caterpillar, or a blank reference chamber, at 50 ml·min^−1^, controlled by mass-flow valves (Sierra Instruments, Monterey, California, USA) and a MFC-2 controller (Sable Systems International, Las Vegas, NV, USA). Carbon dioxide and water vapour were quantified in excurrent air with a LiCor 7000 infra-red gas analyser (LiCor, Lincoln, NE, USA). Activity was monitored by an AD-1 infrared activity detector (Sable Systems). Three caterpillars were measured in each run (using a Sable Systems RM-8 multiplexor), where each run included a 1 h acclimation period, 10 min baseline measurement of the reference chamber at the beginning and end of the run, and 40 min of measurement of each caterpillar sequentially after a 10 min wash-out period. Data were acquired by a Sable Systems UI2 interface, drift-corrected to baseline values, and CO_2_ data were corrected for dilution by water vapour (recorded simultaneously by the LiCor7000). To generate resting CO_2_ production for each larva at each temperature, we analysed a 10 min period with no detectable movement, where CO_2_ production was stable.

### Statistical analyses and modelling

Statistical analyses were performed in R 2.13.0 [24] unless otherwise noted. Terms in models were dropped sequentially from maximal models based on statistical significance and Akaike's information criterion (AIC) was used to confirm the improved fit of the reduced model, with a less than two point difference in AIC being the threshold for retaining the simplified model [25]. Mass and date of dormancy were compared among treatment groups using ANCOVA with rearing conditions and source population as factors and date of dormancy as covariate (mass) or by ANOVA with rearing conditions and source population as fixed factors (date of dormancy). Tukey's *post hoc* comparisons for the final model were performed using PROC GLM in SAS 9.1 (SAS Institute Inc., Cary, NC). The energy use above that predicted from the mean that resulted from variability (energy use calculated using monthly mean meteorological data subtracted from that based on hourly meteorological data) was compared between locations by ANCOVA with location as a factor and daily thermal amplitude as covariate, and then separately by location by regressing extra energy use above that predicted by mean temperature against daily thermal amplitude.

### Thermal sensitivity of metabolic rate

We developed generalised equations describing the relationship between temperature and metabolic rate to allow us to determine the impact of origin and rearing conditions on the thermal sensitivity of metabolism, and to use these in a subsequent model of temperature effects on energy consumption (and therefore fitness) in the field. The relationship between carbon dioxide production and temperature for temperatures below the thermal optimum was modelled in R using a maximum likelihood approach to choose appropriate models incorporating metabolic rate, a scaling factor, and a group-specific term describing thermal sensitivity (as in Equation 1). Starting values for coefficients were selected graphically, and the same values were used for every model. Exponential and power-law nonlinear models (both with and without body mass as a covariate, see Table 1) were fitted to all metabolic rate data using the gnls() function without rearing treatment or source population membership defined, and with a variance structure that assumed variance increased as a power of the temperature [26]. In organisms engaging in minimal metabolic control, metabolic rate does not decline to zero with decreasing temperature but instead converges upon a minimum value that is independent of both temperature and size across taxa [27]. Thus, all models were specified with a final coefficient representing this minimal life supporting metabolic rate (“L_met_”) for *E. propertius* as an additional term, which was estimated from the full set of metabolic rate-temperature data. The best-fitting model was selected based on Akaike's information criterion (AIC) and likelihood ratio tests [28]. The first exponential model investigated (Model 1; Table 2) represents a model based on the metabolic theory of ecology, a commonly postulated model relating metabolic rate to temperature and body mass [10], but this model was not a good fit for our data. The model with the lowest AIC included body mass (Model 4, Table 2); however a likelihood ratio test showed no significant difference in model fit between Model 4 and Model 5 (likelihood ratio test, F_1_ = 3.29, p = 0.07) and the coefficient value for body mass was not significantly different from 0, so Model 5 (the simpler model that did not include body mass) was selected for further investigation. The values for minimum life-supporting metabolic rate (“L_met_”) and scaling coefficient (“S”) determined from this model were 1.66×10^−5^±2.85×10^−6^ mL·min^−1^, and 1.78×10^−7^±7.00×10^−8^ mL·min.°C^−1^, respectively. These values were then used for all following models.

**Table 2:** Generalized nonlinear models relating measured *E. propertius* CO_2_ production rate to measured body temperature.

Model number	Model	AIC	Log-likelihood	df	Parameter p-values
1	*_CO2 = S×M_^−0.25^_×e_^Ts×T^_+Lmet_*	−3291.2	1650.6	5	S: <0.001
					T_s_: <0.001
					L_met_: <0.001
2	*_CO2 = S×M_^Ms^_×e_^Ts×T^_+Lmet_*	−3296.9	1654.5	6	S: <0.001
					M_s_: 0.781
					T_s_: <0.001
					L_met_: <0.001
3	*_CO2 = S×e_^Ts×T^_+Lmet_*	−3300.1	1655.0	5	S: <0.001
					T_s_: <0.001
					L_met_: <0.001
4	*_CO2 = S×M_^Ms^_×T_^Ts^_+Lmet_*	−3306.7	1659.4	6	S: <0.001
					M_s_: 0.7979
					T_s_: <0.001
					L_met_: <0.001
5	*_CO2 = S×T_^Ts^_+Lmet_*	−3305.6	1657.8	5	S: <0.001
					T_s_: <0.001
					L_met_: <0.001

All models provided a better fit than a linear model (*CO2 = S×T*; log-likelihood test, p<0.05 in all cases). Model 5 was selected because there was no significant difference in explanatory power between Models 4 and 5 (likelihood ratio test, L = 3.06, p = 0.08) and Model 5 has fewer terms. A power of the mean model for variance was used in all models. Empirical input: CO_2_: CO_2_ production rate, ml·min^−1^; T: Temperature, °C; M: body mass, mg. Coefficients estimated by maximum likelihood: S: an overall scaling factor; M_s_: a mass scaling factor; T_s_: a temperature scaling factor (i.e. thermal sensitivity). L_met_: theoretical minimum life-supporting metabolic rate. AIC: Akaike's Information Criterion; df: degrees of freedom.

The overall model from the first step was used to investigate model structure and the effect of incubator temperature regime and geographical origin on the metabolic rate reliance on temperature, and used the parameter estimates for L_met_ and S from the previous step to estimate group-specific temperature sensitivity (T_s_). Generalized nonlinear and nonlinear mixed-effects models were fitted to carbon dioxide production rate data using the gnls() and nlme() functions respectively in R (Tables 3 and 4; [29]). Because it was expected that there might be individual effects on this relationship (so the value of T_s_ might vary significantly between individuals), generalized nonlinear mixed effects models were initially investigated. However, 95% confidence interval plots of individual values of these coefficients indicated few differences between individuals, so models without mixed effects were also investigated. Mixed effects models were specified with either T_s_ as a random effect or not. All generalized nonlinear models were given a variance structure whereby variance increased as a power of mean temperature [26]. The estimated power functions were 0.956 and 0.701 for autumn and spring respectively, which indicated that the power of the mean variance function was appropriate [25]. Residual plots indicated homoscedasticity. The overall best-fit models for both time points based on AIC and likelihood ratio tests were generalized nonlinear models with larvae from BC raised at stable temperatures as one group, and all other larvae pooled in a second group (Tables 3 and 4). The extracted experimental group coefficients (Table 5) were used for all subsequent modelling of metabolic rate as a function of temperature. The overall best-fit models for both time points were power-law models with larvae from BC raised at stable temperatures as one group, and all other larvae pooled in a second group (Tables 3 and 4). Coefficients from these models were used to predict energy consumption from meteorological data.

**Table 3:** Comparison of pre-winter thermal sensitivity of CO_2_ production by *Erynnis propertius* among treatment groups.

Model type	Random effects	Experimental groups	AIC	Log-likelihood	df	Coefficient p-value
GNME	T_s_	All rearing conditions×source populations	−1741.8	874.9	4	<0.001
GNME	T_s_	None (all data pooled)	−1725.2	866.6	4	<0.001
GNME	T_s_	BC stable separate, other 3 groups together	−1744.8	876.4	4	<0.001
GN	N/A	All rearing conditions×source populations	−1750.0	881.0^a^	6	<0.001
GN	N/A	None (all data pooled)	−1727.2	866.6^b^	3	<0.001
**GN**	**N/A**	**BC stable separate, other 3 groups together**	**−1752.1**	**880.1^a^**	**4**	**<0.001**

All models use the general form of Model 5 (Table 2). An overall scaling factor (S) and minimum life-supporting metabolic rate (L_met_) were estimated from Model 5, while temperature-scaling (T_s_) was estimated for the experimental groups separately or together. GNME: Generalized nonlinear Mixed Effects model (with T_s_ and S as fixed effects). GN: Generalized Nonlinear model. AIC: Akaike's Information criterion. df: degrees of freedom. The model with the lowest AIC is in bold type, and coefficients from this model were used in subsequent analyses. Different superscript letters indicate generalized nonlinear models that significantly differ in explanatory power (p<0.05). Coefficient p-value is the probability that T_s_ differs significantly from 0.

**Table 4:** Comparison of post-winter thermal sensitivity of CO_2_ production by *Erynnis propertius* among treatment groups.

Model Type	Random effects	Experimental groups	AIC	Log-likelihood	df	Coefficient p-value
GNME	Ts	All rearing conditions×source populations	−1604.6	806.3	4	<0.001
GNME	Ts	None (all data pooled)	−1601.2	804.6	4	<0.001
GNME	Ts,	BC stable separate, other 3 groups together	−1605.5	806.7	4	<0.001
GN	N/A	All rearing conditions×source populations	−1610.5	811.3^a^	6	<0.001
GN	N/A	None (all data pooled)	−1603.3	804.6^b^	4	<0.001
**GN**	**N/A**	**BC stable separate, other 3 groups together**	**−1611.5**	**809.8^a^**	**4**	<0.001

All models use the general form of Model 5 (Table 2). An overall scaling factor (S) and minimum life-supporting metabolic rate (L_met_) were estimated from Model 5, while temperature-scaling (T_s_) was estimated for the experimental groups separately or together. GNME: Generalized nonlinear Mixed Effects model (with T_s_ and S as fixed effects). GN: Generalized Nonlinear model. AIC: Akaike's Information criterion. df: degrees of freedom. The model with the lowest AIC is in bold type, and coefficients from this model were used in subsequent analyses. Different superscript letters indicate generalized nonlinear models that significantly differ in explanatory power (p<0.05). Coefficient p-value is the probability that T_s_ differs significantly from 0.

**Table 5:** Effects of thermal variability on the estimated coefficient of thermal sensitivity (Ts) in *Erynnis propertius*.

Group membership	Time of year	Thermal sensitivity coefficient
BC stable	Autumn	2.54±0.029
All other rearing×source	Autumn	2.41±0.018
BC stable	Spring	2.38±0.025
All other rearing×source	Spring	2.46±0.011

Group-specific coefficients for thermal sensitivity (Ts) from the best-fit generalized nonlinear model (*CO_2_ = S×T^Ts^+L_met_*, Tables 2 & 3) relating *E. propertius* CO_2_ production rate to body temperature, in individuals from a stable environment reared at stable temperatures (BC stable) or individuals originating from a variable environment and/or reared at variable temperatures (all other rearing×source). Value indicated is mean ± SE.

### Meteorological data

Hourly thermal data were obtained from the meteorological stations closest to the collection locales: Victoria International airport, Victoria, BC, Canada and Rogue Valley International-Medford airport, Medford, OR, USA (Table 1, Fig. 2). These data were used as a proxy for microclimate temperatures. Years where any month was missing more than 10% of the hourly observations were excluded (Table 2), and other missing values were replaced with the hourly means for the month in question (where available). When data for an hour were missing for an entire month (1500 h and 2300 h in April 2000 and July 1999 for Vancouver Island), those values were replaced with the mean temperature for that day and time from the complete dataset for that location. Meteorological data were obtained from Environment Canada (Toronto, ON, Canada) or the National Climatic Data Centre (Asheville, North Carolina, USA).

### Overwinter energy consumption

To estimate the energetic costs during dormancy in each environment, we used these data to predict overwinter energy use from 1973 to 2010. Using more than 30 years of climate data prevents our results from being driven by extreme events, unusual years, or climate oscillations, and provides an estimate of the inter-annual variance of energy use at each location across most of the IPCC standard reference period. Thus, although we make empirical measurements on only a few populations and rearing conditions, we are able to explore the expected energetic consequences of observed metabolic regulation over three decades at each location.

Carbon dioxide production for every hour of each winter (from the autumn to the spring equinox) from 1973–2010 was calculated for each group of larvae from hourly or monthly mean climate data from OR and BC using the general equation from Model 5 (Table 2) using a method described previously [30]. Total CO_2_ production per winter was calculated under stable (BC) and variable (OR) thermal regimes for larvae expressing high or low thermal sensitivity. CO_2_ production was converted into lipid used assuming a respiratory exchange ratio (RER) of 0.7 (indicating complete reliance on lipid catabolism), and that 2 mL O_2_ is consumed per mg of lipid catabolised [31]. Comparisons among locations were made with Welch's t-tests, ANOVA and ANCOVA. Unless stated otherwise, an overwinter period (encompassing the duration of dormancy) in our analyses runs from the autumnal equinox of one year to the vernal equinox of the following year. Thus, the 2008 overwinter period ran from September 2008 to March 2009.

To examine the effect of phenology changes on overwinter energy use, we moved the start and end dates of the dormant period forward and backward at daily intervals from the autumn and spring equinox respectively to the most extreme date of dormancy or emergence observed for either population. We then calculated the CO_2_ production for each resulting winter, for every combination of start and end dates observed in the lab and the field for this species (see “Estimates of site-specific phenology”). We took the average of 1973–2010 values for each combination of dates at each site to examine the impact of phenological shifts. Thus, each data point represents the average energy used between that particular start and end date of dormancy combination, calculated from thirty seven years of meteorological data.

### Estimates of site-specific phenology

Date of entry into dormancy was obtained from the laboratory portion of this study (Table 2), while the end of winter was estimated from a combination of field and lab data. Average first flight dates were April 12 and April 3 respectively for BC and OR, and average last sightings were on June 19 and June 14 respectively (derived from transect-based population surveys [20], [32]. Adult *E. propertius* eclose approximately 17 days after transfer to permissive conditions (16L∶8D, 20°C), and average adult lifespan is 28 days when provided with sucrose solution *ad libitum* in the laboratory (CMW, unpublished data). Thus energy use was calculated from 17 days before the first adult sighting to 45 days before the last adult sighting, giving end of winter dates ranging from March 17–May 10.

### Assumptions of the model

Our first assumption in our prediction of energy use from temperature is that carbon dioxide production is a reasonable proxy for metabolic rate. Carbon dioxide production will not capture metabolic processes, such as anaerobic metabolism, which do not result in the production of carbon dioxide, and some insect species do show a shift towards anaerobic metabolism during diapause [4]. This would result in an underestimation of energy consumption, but provided the treatment groups did not differ in their proportion of anaerobic metabolism this will affect only absolute energy use values and not the relationships among groups and locations. We have no reason to believe that our treatment groups would differ in their propensity for anaerobic metabolism.

Our second assumption concerns the relationship between carbon dioxide produced and lipid consumed. We have assumed a respiratory exchange ratio of 0.7 mol O_2_ produced for each mole of oxygen consumed, the ratio observed in animals relying solely on lipid metabolism [31]. Fuel use during diapause is dynamic and, in addition to lipid, insects may also consume carbohydrate, protein, or a mixture of these components; the importance of which can change over the course of the winter [4]. Respiratory exchange ratios for these substrates range from 0.8–1. We also ran all analyses using a respiratory exchange ratio of 1, and our conclusions about relationships among groups were unchanged. Total lipid use estimates are 30% lower if the respiratory exchange ratio was 1, thus our values represent maximum lipid use. If respiratory substrate changes over the course of the winter from lipid to pure carbohydrate (an RQ of 1; [31]), as happens in some overwintering insects [4], our energy use estimates could thus drop by as much as 30%. However, we have no reason to expect such a shift to occur differentially in our treatments. The cost of variability decreases from 0.6 mg to 0.42 mg per degree of daily thermal amplitude assuming a respiratory exchange ratio of 1. We have no reason to believe that treatment groups would differ in their substrate utilisation, although different respiratory exchange ratios among groups would introduce systematic bias. In addition, we note that *E. propertius* stores less than 1/6 as much carbohydrate compared to lipid (CMW, unpublished data), so it is reasonable to assume that lipid is the main fuel for overwintering.

## Results

Measurements in November represent the condition of animals prior to winter, but after a variable period of post-feeding dormancy (which we include in our estimates of overwinter energy consumption). Rearing under variable conditions resulted in larger larvae in November than rearing under stable conditions regardless of source (F_1,109_ = 11.9, p<0.001), and BC larvae were smaller than those from OR when reared under stable conditions (F_1,109_ = 7.1, p = 0.008; Table 6). There was a positive relationship between mass and date of dormancy for larvae reared at variable temperatures (F_1,61_ = 12.8, p<0.001), but not those reared at stable temperatures (F_1,50_ = 1.1, p = 0.304; dormancy date×rearing conditions: F_1,109_ = 18.9, p<0.001). Thus, extended post-feeding dormancy in variable conditions resulted in smaller larvae in November. Larvae kept at variable temperatures entered dormancy 3–4 weeks earlier than those kept at stable temperatures (F_1,112_ = 267.8, p<0.001, Table 6), leading to an increased period of post-feeding energy drain in the autumn. Under variable conditions, BC larvae entered dormancy significantly earlier than those from OR (F_1,112_ = 14.7, p<0.001; Table 6), and thus experienced increased post-feeding energy drain.

**Table 6:** Life history, physiological and experimental design parameters of *Erynnis propertius* larvae used in source×rearing conditions common-garden experiments.

Source	BC		OR	
Rearing	Stable	Variable	Variable	Stable
**N_mothers_**	37	49	14	22
**N_dormant_**	32	35	28	20
**Date of dormancy**	Sep 4±6.3 days^c^	Aug 9±7.9 days^b^	Aug 17±8.7 days^a^	Sep 7±7.6 days^c^
**Mass (mg)**	210.5±34.8^c^	244.1±52.3^a^	246.5±43.4^a^	231.3±31.7^b^
**Lipid (mg·mg DM^−1^)**	0.129±0.030^b^	0.099±0.026^a^	0.090±0.028^a^	0.130±0.026^b^
**Estimated total lipid (mg)**	8.1	7.0		9.0

N_mothers_: the minimum number of mothers from which larvae for each group originated. N_dormant_: number of larvae surviving to enter dormancy. Lipid concentrations and mass were measured in November, after the post-feeding, pre-winter period in autumn. DM: dry mass. Values for date of dormancy, mass and lipid concentration are mean ± SD, different superscript letters indicate significant differences among treatment groups.

**Figure 4:**
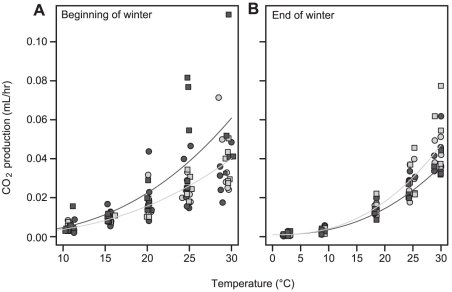
Thermal sensitivity of CO_2_ production in *Erynnis propertius* at the beginning and end of winter. Temperature-CO_2_ production relationships of *E. propertius* larvae measured at (a) the beginning (n = 24) or (b) the end (n = 24) of winter. Dark grey indicates larvae originating from BC, light grey shading those from OR. Circles indicate variable, squares stable, rearing temperature conditions. The dark grey line shows the best model fit for larvae from BC reared at stable conditions; the light grey line the best model for the other three treatments.

Larvae reared under stable conditions had higher lipid concentrations than their variable-reared counterparts in November (F_1,15_ = 7.8, p = 0.014; Table 6), indicative of reduced energy drain in the pre-winter period. Estimated total lipid content was lowest in variable-reared larvae from both source populations, intermediate in BC/stable larvae, and highest in OR/stable larvae (Table 6). In November, immediately after the most variable temperatures were experienced, metabolic rate was depressed at high temperatures in all OR-sourced individuals, and in BC larvae reared under variable conditions, significantly decreasing thermal sensitivity relative to stable-reared BC larvae (Fig. 4a, Tables 3, 4, 5). The suppressed metabolic rates would serve to decrease post-feeding energy drain in those groups that express it. In February, when the larvae were ready to resume development, thermal sensitivity was slightly but significantly higher in variable-reared and OR/stable larvae compared to the BC/stable animals (Fig. 4b).

**Figure 5:**
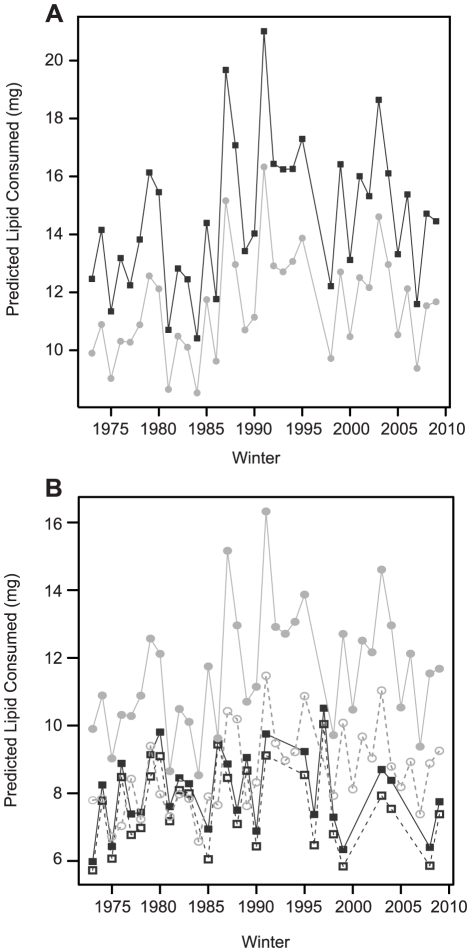
Predicted overwinter energy use of *Erynnis propertius* from Oregon and Vancouver Island. (a) Predicted overwinter energy use from 1973–2010 in Oregon for larvae expressing high (dark squares) or low (light circles) thermal sensitivity. (b) Predicted energy use in their natal environment for larvae expressing high (BC, dark grey squares) or low (OR, light grey circles) thermal sensitivity, based on either hourly (solid lines, filled symbols) or monthly mean (dotted lines, open symbols) temperatures.

**Figure 6:**
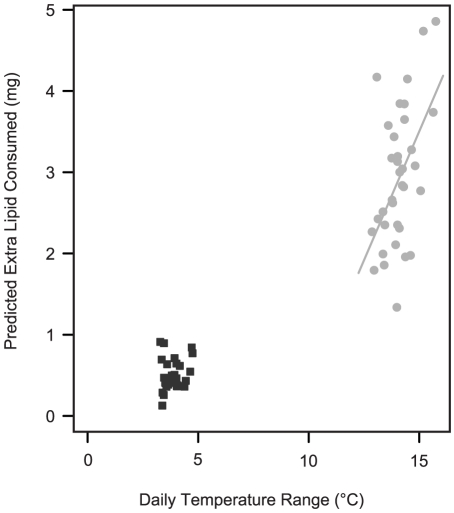
The predicted energetic cost of thermal variability for overwintering *Erynnis propertius*. Predicted extra lipid used overwinter by *E. propertius* larvae above that predicted by mean temperatures as a function of daily thermal amplitude in a given winter in OR or BC. Dark symbols: larvae with high thermal sensitivity in BC; light symbols: larvae with low thermal sensitivity in OR.

To predict the consequences of metabolic suppression on overwinter (inter-equinox) energy use by larvae from variable environments, we used the equations describing the temperature-metabolic rate relationship for each source population, and thirty-seven years of meteorological data from Oregon, which allows us to examine the generality of our conclusions over a long time period. Larvae expressing the low thermal sensitivity phenotype were predicted to consume approximately 20% less energy compared to those with high thermal sensitivity (Fig. 5a). We calculated inter-equinox energy use for each source population in their natal environment, and predicted energy use on Vancouver Island to be lower than in Oregon, based on both hourly temperatures (i.e. incorporating thermal variability; t_59.2_ = 8.7, p<0.001) and monthly mean temperatures (t_56.3_ = 3.11, p = 0.003, Fig. 5b). The magnitude of difference between the locations was greatest and more consistent when variability was accounted for (Fig. 5b). We subtracted the consumption predicted from mean temperatures from that calculated from hourly temperatures, and found that the additional energetic cost of thermal variability over a six month winter was expected to be greater in variable than stable conditions (F_1,58_ = 15.14, p<0.001). In the variable climate, the expected energetic cost of thermal variability was 0.6 mg lipid/winter for every degree of additional temperature range (F_1,33_ = 11.15, p = 0.002, Fig. 6). There was no relationship between the predicted energetic cost of thermal variability and daily thermal range in the stable environment (F_1,25_ = 1.20, p = 0.284, Fig. 6).

**Figure 7:**
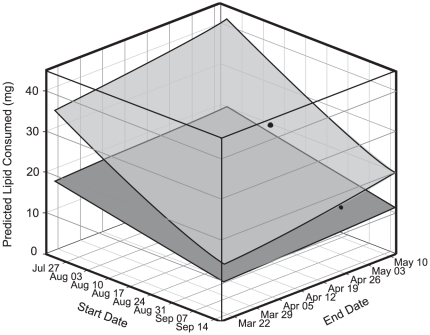
The predicted impact of phenological shifts on overwinter energy use by *Erynnis propertius*. The sensitivity of overwintering energy use of *E. propertius* larvae to phenological shifts in OR (low thermal sensitivity; light grey) or BC (high thermal sensitivity; dark grey). Dates encompass the full range of start and end times of dormancy in *E. propertius* (Table 2), and each surface represents the average of 37 years' energy use at that location. Black dots indicate location-specific start and end dates; from the average date of dormancy onset to median date of adult flight.

We used our hourly estimates of lipid use in each location (1973–2010) to predict the energetic impact of phenological shifts at the beginning and end of the dormant period. At any given length of dormancy, overwintering in stable Vancouver Island conditions is less energetically expensive than overwintering in variable Oregon conditions (Fig. 7), despite the metabolic rate suppression expressed by OR-derived individuals. This discrepancy in energetic costs between locations increases with longer dormant periods, and phenological changes have more impact in variable conditions (Fig. 7). Using start and end dates informed by field observations to establish a ‘typical’ phenology, we predict lipid consumption in OR (27.3 mg) to be more than double that on BC (12.7 mg; Fig. 7). Our models indicate a steeper decline in energy use with delayed diapause date compared to advancing emergence date (Fig. 7), suggesting that phenological shifts will have a greater energetic impact in autumn than in spring.

## Discussion

Here we show that phenological shifts will have more impact on energy use by ectotherms in variable thermal environments, that the impact will be more severe in the autumn, and that this vulnerability persists even when organisms have adaptations that reduce thermal sensitivity of metabolism in response to thermal variability. Variation in phenology is heritable and under strong selection in many species [33], and phenological shifts have been among the best-documented consequences of recent climate change [34]. We would therefore expect populations from variable environments to be selected for delayed entry into dormancy, but *E. propertius* from OR reared at variable temperatures actually enter dormancy almost three weeks before stable-reared larvae. Larvae reared under variable conditions are larger than those in stable environments, so the lack of delay could result from larvae entering dormancy at a pre-determined instar or size threshold [35] that is reached earlier in the more variable environment due to higher metabolic and growth rates [36]. Early dormancy could also be driven by declining host plant quality early in the autumn in variable environments [37] (although there were no such cues in our laboratory rearing). When raised in a variable thermal environment, larvae from BC enter dormancy even earlier than larvae from OR. This suggests that OR larvae may have been selected to delay the onset of dormancy, perhaps to mitigate the deleterious effects of high autumn temperatures.

Our results provide empirical support for theoretical predictions of a decrease in the thermal sensitivity of performance functions in response to increasing temporal variability [8]. We show metabolic suppression in overwintering caterpillars in response to daily thermal variability, which appears to be facultative in the BC population. By contrast, all larvae of OR origin expressed the suppressed metabolism phenotype, suggesting that metabolic suppression is genetically fixed in that population. Biochemical costs of decreased thermal sensitivity may precipitate trade offs against other traits [38], for example overwinter immune function or developmental or reproductive latency, which would maintain the facultative response in the BC population. Thus, thermal variability outside the growing season appears to select for a metabolic depression phenotype. Climate change could lead to increases in thermal variability, or range expansion could shift species to more variable environments. We would expect these changes to negatively impact overwintering ectotherms which lack the ability to suppress their overwinter metabolic thermal sensitivity.

Dormant insects are small-bodied, with limited thermoregulatory capabilities, and thus experience fluctuations in body temperature throughout the day that make mean temperatures a poor estimate of metabolic expenditure. If mean temperatures are used (effectively ignoring Jensen's inequality), the predicted impact of thermal variability on energy use is lost because the estimates of metabolic rate no longer incorporate the unequal impacts of high temperatures. For example, in some years the mean conditions predict lower energetic costs in the variable environment than in the stable environment, but when we account for the thermal variability revealed by hourly data, the stable environment is always less energetically demanding. The relative differences in energetics may be exacerbated in thermally variable years if BC larvae facultatively suppress thermal sensitivity of metabolism in the field. We believe that our approach of using hourly thermal data, which better incorporates the non-linear effects of temperature on metabolic rate, will improve the accuracy of predicting whole-insect performance measures such as development time compared to degree-day models [39], and may be more generally applicable to other non-feeding dormant temperate ectotherms, as well as torpor-phase hibernating mammals.

Our models were driven by climate rather than microclimate temperature, and thus assume that the caterpillars were experiencing conditions equivalent to air temperature two meters above the ground. As *E. propertius* overwinters on the ground, the main modifiers of microclimate temperature will be leaf litter and snow [40]. Leaf litter may buffer variability to some extent, decreasing the highs and increasing the lows compared to air temperature without substantially changing the mean [41], meaning our absolute values may be slightly overestimated. However, we assume that leaf litter will buffer microclimates to similar degrees in both locations. Snow cover generally increases temperature relative to ambient by preventing temperatures from dropping below zero, and so would be expected to increase energy use. However, snow cover is not extensive in these habitats in either Oregon or British Columbia. Ideally, microclimate temperatures would be used to more accurately approximate the operative temperature of the animals.

Despite the energy savings afforded by decreased thermal sensitivity, the variable environment was still more energy demanding – in a ‘typical’ winter, larvae in the variable environment were predicted to have double the energy expenditure of those in more stable temperatures. Variable-reared larvae were larger than their stable-reared counterparts (and OR larger than BC, see also [21]), but a longer period of dormancy in autumn led to decreased lipid concentrations and contents, especially in those larvae that experienced variable conditions. This supports our model prediction of increased energy drain in autumn that negates the potential advantages of larger size and decreased thermal sensitivity in OR or stable-reared larvae. Thus, the predisposition to increased size is consistent with selection for greater energy stores in response to daily thermal variability in autumn [42]. The total lipid quantity in November is low, but consistent with our model predictions of high autumn and negligible post-autumn lipid consumption. Thus, future studies determining energy reserves available for spring reproduction in overwintering ectotherms should account for autumn energy consumption, and how it may differ among locations.

Our model shows that thermal variability explains why early entry into dormancy decreases fitness of overwintering insects, while extended cool springs have less impact [18], [19]. Thus, although phenology research has focused on spring metrics such as first flight, flowering, or breeding dates (e.g. [43]), we show that it is important to thoroughly document autumn phenological events such as leaf drop, migration, and entry into dormancy because of the significant potential impact on overwinter energetics. Although in our study system the centre of the range (OR) is more variable than the northern range edge (BC) due to oceanic influences on Vancouver Island, daily thermal range typically increases with increasing latitude and elevation [44]. Therefore, species following isotherms upwards or polewards in response to climate change may encounter changes in variability leading to increased energetic demands. In addition, predicted increases in mean temperatures [45] will shift daily thermal cycles to fluctuate at a point where the metabolic rate-temperature relationship is steeper, compounding energetic drain [11]. If overwinter energy consumption increases, this could precipitate life-history trade-offs such as decreases in adult size and fecundity that could lower the fitness of individuals and increase likelihood of population declines in many temperate, univoltine, insects, and possibly other temperate ectotherms as well.
